# Case report: acute abdominal pain in a 37-year-old patient and the consequences for his family

**DOI:** 10.1186/s12876-020-01283-2

**Published:** 2020-05-03

**Authors:** Elisabeth Niemeyer, Hamid Mofid, Carsten Zornig, Eike-Christian Burandt, Alexander Stein, Andreas Block, Alexander E. Volk

**Affiliations:** 1 Department of Medical Oncology and Hematology, University Cancer Center Hamburg, University Hamburg-Eppendorf, Hamburg, Germany; 2 Department of Surgery, Regio Klinikum Pinneberg, Pinneberg, Germany; 3 Israelitisches Krankenhaus, Hamburg, Germany; 4 Institute of Pathology, University Medical Center Hamburg-Eppendorf, Hamburg, Germany; 5 Institute of Human Genetics, University Medical Center Hamburg-Eppendorf, Hamburg, Germany

**Keywords:** Hereditary diffuse gastric cancer, *CDH1* germline mutation, Prophylactic gastrectomy

## Abstract

**Background:**

Hereditary diffuse gastric cancer is a rare condition that accounts for approximately 1–3% of all gastric cancer cases. Due to its rapid and invasive growth pattern, it is associated with a very poor prognosis. As a result, comprehensive genetic testing is imperative in patients who meet the current testing criteria in order to identify relatives at risk. This case report illustrates the substantial benefit of genetic testing in the family of a patient diagnosed with hereditary diffuse gastric cancer.

**Case presentation:**

A 37-year-old patient was admitted to the emergency department with acute abdominal pain. Following explorative laparoscopy, locally advanced diffuse gastric cancer was diagnosed. The indication for genetic testing of *CDH1* was given due to the patient’s young age. A germline mutation in *CDH1* was identified in the index patient. As a result, several family members underwent genetic testing. The patient’s father, brother and one aunt were identified as carriers of the familial *CDH1* mutation and subsequently received gastrectomy. In both the father and the aunt, histology of the surgical specimen revealed a diffuse growing adenocarcinoma after an unremarkable preoperative gastroscopy.

**Conclusion:**

Awareness and recognition of a potential hereditary diffuse gastric cancer can provide a substantial health benefit not only for the patient but especially for affected family members.

## Background

Gastric cancer is the fifth most commonly diagnosed malignancy and the third most deadly cancer in males worldwide [1]. Gastric adenocarcinoma can be classified according to Lauren’s criteria, which define two major histological subtypes: intestinal and diffuse type adenocarcinoma [2]. These two subtypes have a variety of distinct clinical and molecular pattern. While the more common intestinal type of adenocarcinoma has a stronger association with Helicobacter pylori infection and dietary risk factors, diffuse gastric cancer more often has a genetic etiology [3]. Intestinal type of gastric cancer is frequently preceded by long-standing, precancerous, ulcerous lesions and is easily detectible by gastroscopy [4]. In contrast, diffuse gastric cancer typically develops within the submucosa with small foci of signet cells dispersed throughout the tissue, often not easily detected by routine upper endoscopy and can be missed in superficial biopsies [5]. Diffuse gastric cancer is typically associated with an aggressive growth pattern and poor prognosis [3].

Germline mutations in the *CDH1* gene, encoding E-cadherin, are associated with an autosomal-dominant inherited susceptibility for diffuse gastric cancer (hereditary diffuse gastric cancer/HDGC [Online Mendelian Inheritance in Man®/OMIM entry #137215). Mutations in *CDH1* can be identified in up to 54% of HDGC cases by sequence analysis and gene-targeted analysis for deletion and duplication [6] Although rare, other genetic causes of HDGC are still under investigation, including mutations in the CTNNA1 gene and mutations in other genes [7]. Invasion of carcinomas into surrounding tissues and their eventual metastasis requires the process of epithelial-mesenchymal transition (EMT). Downregulation or dysfunction of E-cadherin is the hallmark event in EMT, as E-cadherin deficient cells lose their ability to adhere to each other and gain individual cell motility [8]. In the case of HDGC, E-cadherin deficiency leads to a diffuse growth pattern of cancer cells throughout the submucosa. The gastric mucosa is intact and appears normal during gastric endoscopy even in late disease [5]. Loss of expression of E-cadherin in immunohistochemistry may be an indicator for a *CDH1*-associated gastric cancer. The cumulative life time risk for carriers of a pathogenic variant in *CDH1* to develop gastric cancer is 70% for men and 56% for women [5, 7]. Women carrying a *CDH1* mutation also have an increased risk for breast cancer, especially lobular breast cancer (42% lifetime risk with an average age of onset of 53 years [6]). It is still unresolved whether *CDH1*-mutation carriers also have an increased risk of colon cancer [5].

## Case presentation

**Fig. 1:**
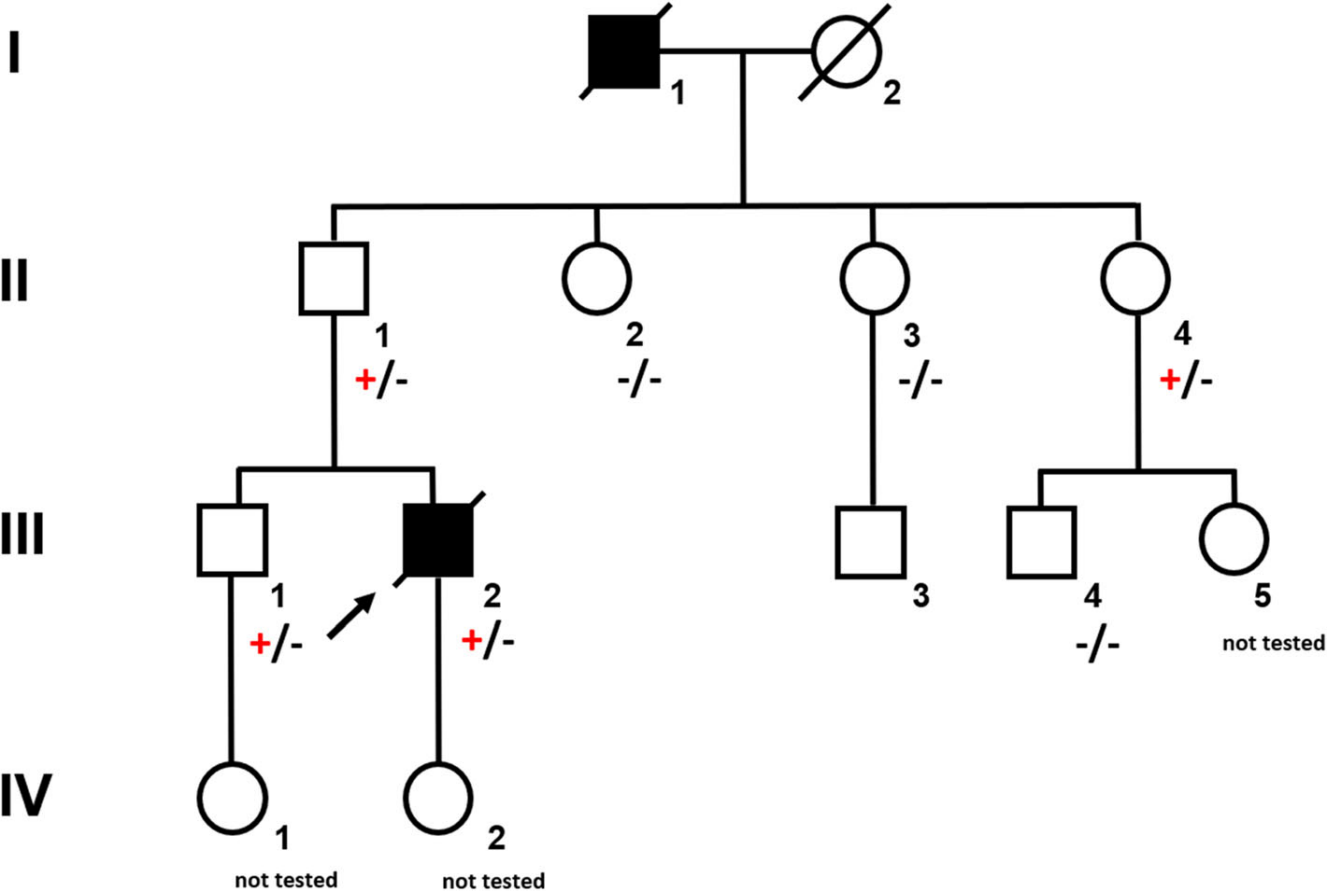
Pedigree of a large German family segregating autosomal dominant *CDH1*-associated gastric cancer. Circles represent females and squares males. Filled symbols indicate clinically affected individuals and a plus sign carriers of the *CDH1* mutation c.1137G > A

A 37-year-old male (Fig. 1, III.2) was referred to the emergency room with acute abdominal pain and fever. Physical examination showed signs of an acute peritonitis and lab tests revealed elevated inflammation markers (C-reactive protein). CT-scan showed a pneumoperitoneum and an emergency exploratory laparoscopy was performed. Since the site of the perforation could not be detected by laparoscopic approach, the procedure had to be converted to an open laparotomy. A gastric perforation (2 mm in diameter) was found, excised and sutured. Unexpectedly, the surgeons noted a rigid thickened stomach wall (consistent with linitis plastica) during operation. Furthermore, a tumor mass invading the minor omentum and the mesentery was found. Intra-surgical frozen section analysis confirmed the initial suspicion and revealed diffuse growing gastric cancer with signet cells. Finally, the patient was diagnosed with a diffuse gastric cancer (pT4bN3M1).

**Fig. 2:**
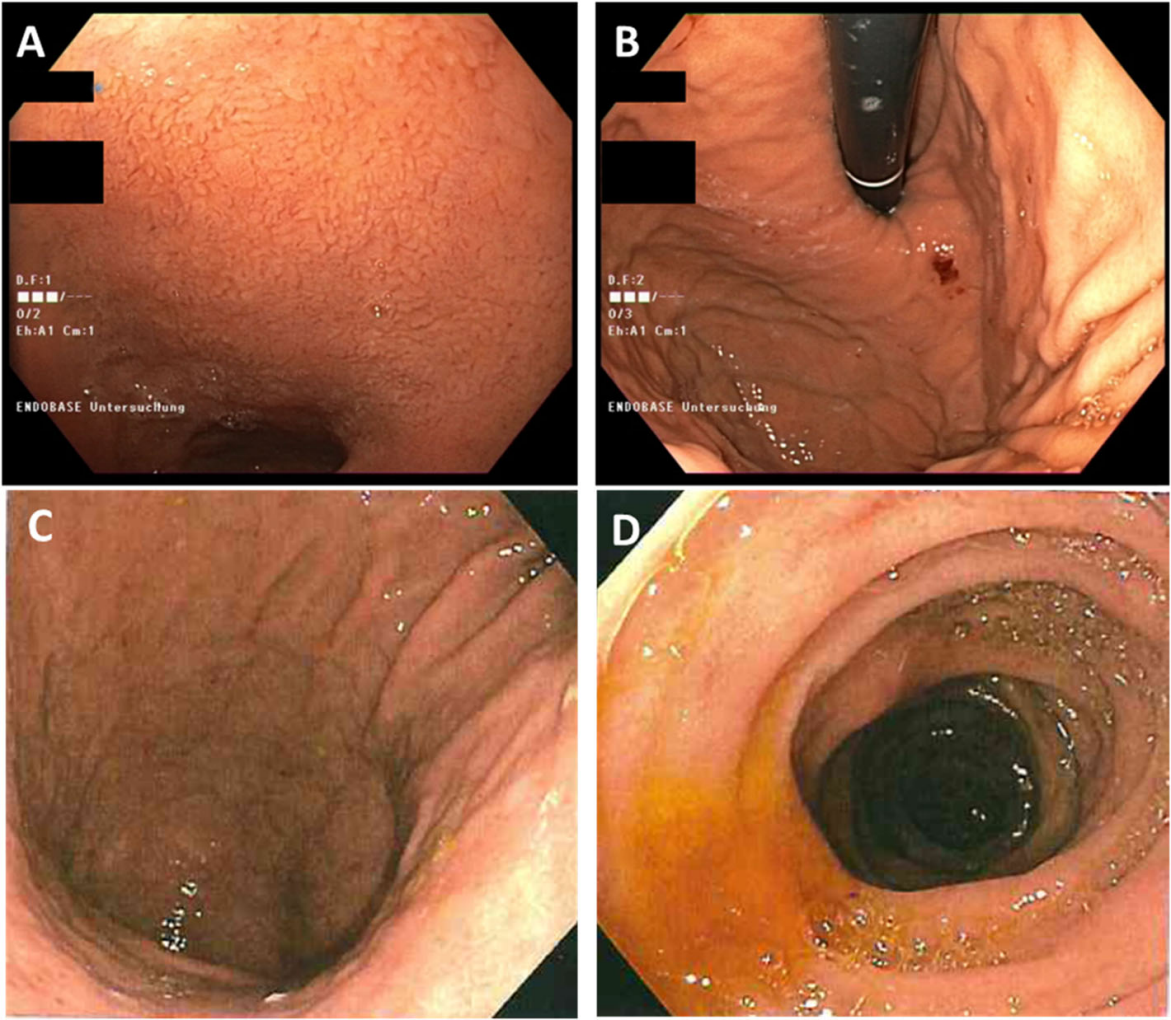
Endoscopic images of the paternal aunt (**a/b**) and the father (**c/d**) showing normal appearance of the gastric mucosa despite later confirmed signet cell adenocarcinoma in the submucosa

**Fig. 3:**
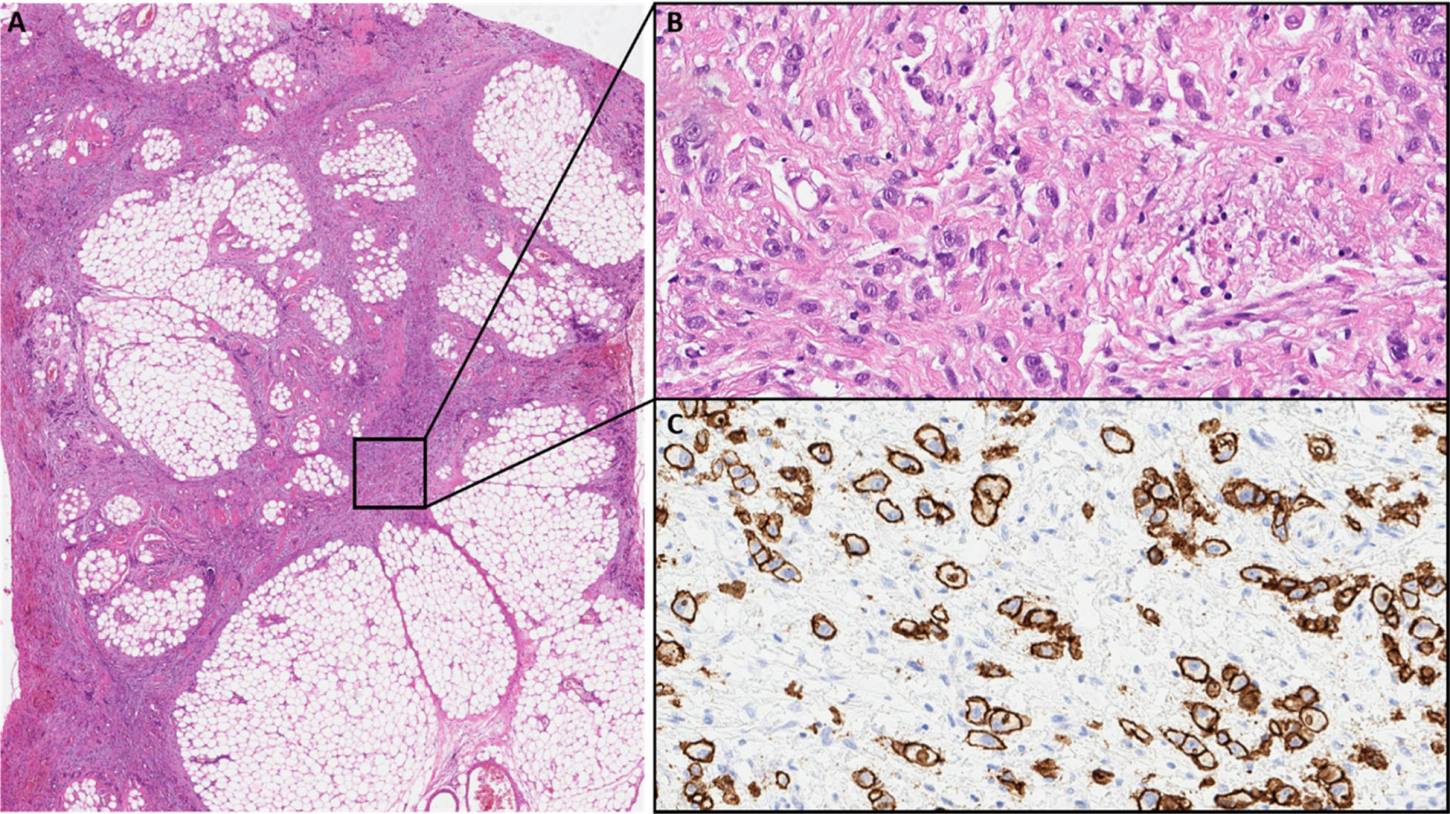
**a** Low power view of perigastric fat infiltrated by the gastric cancer (HE). **b** High power view showing diffuse growing isolated cancer cells (HE). **c** High power view of an E-cadherin immunohistochemistry stain with Antibody: Agilent Dako FLEX Monoclonal Mouse Anti-Human E-Cadherin, Clone NCH-38 showing strong membranous positivity of the cancer cells. With knowledge of the molecular data the E-cadherin antibody most probably detects a non-functional E-cadherin protein

The young age at diagnosis and histological subtype prompted the surgeon to refer the patient and his family to genetic counselling. Evaluation of the family history revealed that the patient’s paternal grandfather (Fig. 1, I.1) died of abdominal cancer at the age of 40. No other family members suffered from cancer, especially not the father (61 years old; Fig. 1, II.1). Both the father and the healthy older brother (Fig.1 III.1) underwent elective gastroscopies. In both patients, endoscopy showed an unremarkable mucosa (Fig. 2c/d, data for brother not shown). Multiple random “button hole biopsies” were taken during gastroscopy and diffuse growing malignant cells could be detected in one out of three of the biopsies taken from the father. Genetic testing revealed the heterozygous germline mutation c.1137G > A of *CDH1.* This genetic alteration affects the last exonic nucleotide at the canonical splice donor site leading to impaired splicing and consequently to a dysfunctional E-cadherin molecule (Human Gene Mutation Database/HGMD® No. CS0060517). As this mutation has already been described in other HDGC patients, the diagnosis of HDGC in the father and also in the index patient carrying the same mutation was confirmed. Surprisingly, despite the presence of a *CDH1* mutation, E-cadherin expression could still be immunohistochemically detected in tumor tissue with a monoclonal antibody raised against E-cadherin (Fig. 3, Agilent Dako FLEX Monoclonal Mouse Anti-Human E-Cadherin, Clone NCH-38).

With identification of a pathogenic *CDH1* mutation, index patient’s healthy brother (38 years old; Fig. 1 III.1) as well as the three paternal aunts (53-, 56-, 58- years old, respectively, Fig. 1, II.2, II.3, II.4) had an a-priori risk of 50% to also carry the *CDH1* mutation. Ultimately, all four relatives underwent predictive testing after genetic counselling. While the mutation was detected in the brother and one aunt (Fig. 1, II.4), two aunts did not inherit the mutation. As a result, his brother and his aunt underwent prophylactic gastrectomy. While no malignant cells were found in the histologic examination of the brother’s stomach, a diffuse growing adenocarcinoma was detected during histologic workup in the stomach of the aunt. Despite a macroscopically unremarkable appearance of the surgical specimen, two isolated cancer foci of signet cells located 7 mm from one another were detected in the upper third of the lamina propria of the cardiac region. Similar to the index patient’s father /herbrother, this diffuse gastric cancer was not macroscopically detectable during gastroscopy (Fig. 2a/b) before gastrectomy was performed, not even after retrospectively analyzing the affected sites in the endoscopic images. Further staging showed no involvement of lymph nodes or distant metastasis in her case (pT1a pN0 (0/16) cM0).

The father, brother and aunt recovered well after their surgeries. Unfortunately, the condition of the index patient worsened quickly due to perioperative complications. Systemic chemotherapy could not be administered due to his poor general condition. He died 2.5 months after diagnosis.

## Discussion and conclusions

Given that monogenic factors are rare and account only for 1–3% of gastric cancers, awareness among health care professionals is of utmost importance. Regardless of the family history, a personal history of a diffuse gastric cancer under the age of 40 qualifies for genetic testing of the *CDH1* gene following the latest International Gastric Cancer Linkage Consortium (IGCLC) consensus guidelines. Furthermore, a *CDH1* mutation should be suspected if two or more family members have been diagnosed with diffuse gastric cancer at any age or in a family if two family members were diagnosed with diffuse gastric cancer or lobular breast cancer with one diagnosis before the age of 50 years (Table 1, [5]). In our family, the young age of the index patient and the family history with abdominal cancer of the paternal grandfather lead to genetic testing and the identification of the causal *CDH1* mutation in the family. With the identification of the underlying genetic defect, all family members had the chance to learn about their exact individual risks. First degree relatives of a mutation carrier have a 50% chance to inherit the mutation. Extensive genetic counseling and discussion of the clinical management is especially important when detecting a mutation by predictive testing in healthy relatives. If a family member has not inherited the mutation, he and his progeny have no increased risk to develop gastric cancer. On the other hand, if the pathogenetic mutation is detected, the individual is faced with the cancer risks previously mentioned. As diffuse gastric cancer is difficult to detect at an early and treatable stage, mutation carriers may either choose prophylactic gastroscopy or endoscopic surveillance according to the Cambridge protocol [9]. This protocol recommends targeted biopsies of any suspicious lesion in the gastric mucosa as well as a minimum of 6 random biopsies taken from each anatomic area of the stomach (antrum, transitional zone, body, fundus, cardia) and should begin 5–10 years prior to the diagnosis of the youngest family member [9, 10]. Current recommendations suggest prophylactic gastrectomy in healthy CDH1 mutation carriers rather than endoscopic surveillance [5]. The estimated risk for diffuse gastric cancer is 1% by the age of 20 years and around 4% by the age of 30 years [10]. Therefore, most authors recommend offering a gastrectomy to CDH1 mutation carriers between ages 20 and 30 [5, 10]. The mortality rate of gastrectomy itself is less than 1% but it has a high morbidity for nutritional, metabolic and psychological well-being. Most patients report rapid intestinal transit, reflux, dumping syndrome and diarrhea after surgery. After gastrectomy patients need life-long vitamin supplementation. In our family, all mutation carriers chose to undergo gastrectomy, and gastric cancer was diagnosed at an early stage in two individuals. In both cases, preoperative endoscopy failed to detect the cancer. However, in random biopsies taken from endoscopically normal gastric mucosa, cancer cells were detected. Considering the overall small tumor size in both patients, the detection of tumor cells through random biopsies was a very fortunate incident for our family and underlines the need for taking a minimum of 30 deep biopsies as recommended in the Cambridge protocol. Despite advanced endoscopic techniques, the overall detection rate through preoperative endoscopic biopsies is low. In 87.9% of *CDH1*-mutation carriers, foci of signet cells were initially detected after prophylactic gastrectomy [11].

**Table 1:** Established criteria for germline *CDH1* testing (family history includes 1st and 2nd degree relatives), [1]

2 gastric cancer cases in a family regardless of age with at least one confirmed diffuse gastric cancer	
1 diffuse gastric cancer before the age of 40	
Personal or family history of diffuse gastric cancer and lobular breast cancer, one diagnosed before the age of 50	

In addition, women carrying a *CDH1* mutation have an increased risk for lobular breast cancer. There is insufficient evidence for a risk-reducing mastectomy, although it might be discussed in individual cases based on the family history [12]. Compared to non-lobular breast cancers, there is a reduced sensitivity to detect lobular breast cancer by mammography [13]. Extrapolated from the data for high-risk hereditary breast cancer, surveillance programs for early detection include e.g. bilateral MRIs beginning at the age of 30 years [12]. Following the recommendation of the German Consortium for Hereditary Breast and Ovarian Cancer (GC-HBOC), our patient was offered annual MRIs and ultrasound of the breast until the age of 70 years. Although it is still unclear whether colon cancer is part of the *CDH1-*related tumor spectrum, we recommended colonoscopies more frequently and at a younger age for all mutation carriers (every 3 years starting at age 40) compared to our current national guidelines (every 5 years starting at age 50).

The diagnosis of diffuse gastric cancer with signet cells will likely prompt the pathologist to an immediate histopathological workup including immunohistochemistry for E-cadherin. However, these steps should be taken with great caution. As the *CDH*1 gene is a tumor suppressor gene, the inactivation of both alleles is necessary for tumor initiation. The inactivation of the second allele in mutation carriers occurs mainly by hypermethylation of the *CDH1* promotor [14]. While hypermethylation itself will result in a silencing of the respective allele and ultimately to a loss of its protein, some mutations give rise to a translated but non-functional protein. Currently, more than 180 pathogenic germline mutations in the *CDH1* gene are listed in HGMD® Professional (2019.4). Most of these are truncating mutations which are predicted to elicit nonsense-mediated mRNA decay [15], which would again result in a loss of the protein. Around 20% of all the published pathogenic alleles are missense mutations which are more prone to result in non-functional, but translated and therefore potentially immunohistochemically detectable protein. The latter could result in an erroneous exclusion of a *CDH1*-associated disease. The mutation identified in our family was shown before in another publication to escape nonsense-mediated mRNA decay and lead to aberrantly spliced RNA [16]. Indeed, E-cadherin expression was immunohistochemically detected in the histopathological workup in the surgical specimen of our index patient and could have led to the misinterpretation of a non-*CDH1-*associated diffuse gastric cancer. Given this assumption, no genetic testing would have been performed, and the underlying genetic cause would not have been unraveled. In the case of our family, this again would have hindered predictive testing for relatives at risk and the identification of two mutation carriers and three non-mutation carriers. Considering the poor prognosis of patients suffering from invasive diffuse gastric cancer, the patient’s father, brother and aunt (and potentially their progeny) had a major benefit from this genetic testing. The index patient’s child and the brother’s child (Fig. 1, IV. 2, IV. 1) were infants at the time of genetic counseling. Predictive testing in individuals at risk younger than 18 years is a controversial issue. Rare cases of hereditary diffuse gastric cancer in individuals before the age of 18 have been reported [17, 18]. It has therefore been suggested to offer predictive testing to individuals before the age of 18 on a case by case basis [5]. The IGCLC has agreed that genetic testing of minors at risk should consider the earliest age of cancer onset in the respective family as well as their physical and psychological resources.

In our case, we decided to evaluate future recommendations for clinical management for families affected by hereditary diffuse gastric cancer and offer genetic counseling to the minors involved in their teens. Because of an independent but severe medical illness, the aunt (III.) and her husband as the legal guardians, decided not to test their daughter. With regard to prenatal testing, there are a number of ethical issues as well as legal provisions to be addressed, especially if testing is being considered for the purpose of pregnancy termination rather than early detection and surveillance. This sensitive issue was thoroughly discussed with the brother of the index patient. The German national legal framework allows invasive prenatal testing in case of a potential treatment during pregnancy or childhood (not applicable in case of *CDH1*-associated diffuse gastric cancer) or in case of an expected disease manifestation before the age of 18 years (subject of interpretation in case of *CDH1*-associated diffuse gastric cancer, see above). During consultation the family ruled out invasive prenatal testing for further family planning due to ethical reasons, although preimplantation genetic diagnosis was an option the family would consider in the future.

In this case of a young patient with sudden death, awareness and recognition of a CDH1-associated hereditary diffuse gastric cancer facilitated the detection of one additional case of early gastric cancer in a family member and most likely prevented two more relatives (and potentially their progeny) from developing gastric cancer, which has a poor prognosis. Therefore, a family history leading to the suspicion of a hereditary cause of diffuse gastric cancer should prompt genetic counseling and low-threshold genetic testing of the *CDH1* gene despite histological expression of E-cadherin.

## Data Availability

n.a.

## Abbreviations

CDH1: Cadherin 1
CT: Computed tomography
HDGC: Hereditary Diffuse Gastric Cancer
IGCLC: International Gastric Cancer Linkage Consortium
(m)RNA: (messenger) Ribonucleic Acid
